# To Help or Not to Help? Prosocial Behavior, Its Association With Well-Being, and Predictors of Prosocial Behavior During the Coronavirus Disease Pandemic

**DOI:** 10.3389/fpsyg.2021.775032

**Published:** 2022-02-11

**Authors:** Elisa Haller, Jelena Lubenko, Giovambattista Presti, Valeria Squatrito, Marios Constantinou, Christiana Nicolaou, Savvas Papacostas, Gökçen Aydın, Yuen Yu Chong, Wai Tong Chien, Ho Yu Cheng, Francisco J. Ruiz, María B. García-Martín, Diana P. Obando-Posada, Miguel A. Segura-Vargas, Vasilis S. Vasiliou, Louise McHugh, Stefan Höfer, Adriana Baban, David Dias Neto, Ana Nunes da Silva, Jean-Louis Monestès, Javier Alvarez-Galvez, Marisa Paez-Blarrina, Francisco Montesinos, Sonsoles Valdivia-Salas, Dorottya Ori, Bartosz Kleszcz, Raimo Lappalainen, Iva Ivanović, David Gosar, Frederick Dionne, Rhonda M. Merwin, Maria Karekla, Angelos P. Kassianos, Andrew T. Gloster

**Affiliations:** ^1^Clinical Psychology and Intervention Science, Department of Psychology, University of Basel, Basel, Switzerland; ^2^Department of Health Psychology and Pedagogy, Riga Stradiņš University, Riga, Latvia; ^3^Kore University Behavioral Lab, Faculty of Human and Social Sciences, Kore University of Enna, Enna, Italy; ^4^Department of Social Sciences, School of Humanities and Social Sciences, University of Nicosia, Nicosia, Cyprus; ^5^Department of Nursing, Cyprus University of Technology, Limassol, Cyprus; ^6^Cyprus Institute of Neurology and Genetics, Nicosia, Cyprus; ^7^Department of Psychological Counseling and Guidance, Faculty of Education, Hasan Kalyoncu University, Gaziantep, Turkey; ^8^The Nethersole School of Nursing, Faculty of Medicine, The Chinese University of Hong Kong, Hong Kong, Hong Kong SAR, China; ^9^Department of Psychology, Fundación Universitaria Konrad Lorenz, Bogotá, Colombia; ^10^Faculty of Psychology, Universidad de La Sabana, Chía, Colombia; ^11^Nuffield Department of Orthopaedics, Rheumatology and Musculoskeletal Sciences, University of Oxford, Oxford, United Kingdom; ^12^School of Psychology, University College Dublin, Dublin, Ireland; ^13^Department of Medical Psychology, Innsbruck Medical University, Innsbruck, Austria; ^14^Department of Psychology, Babes-Bolyai University (UBB), Cluj-Napoca, Romania; ^15^ISPA—Instituto Universitário, APPsyCI—Applied Psychology Research Center Capabilities and Inclusion, Lisbon, Portugal; ^16^Faculdade de Psicologia, Universidade de Lisboa, Alameda da Universidade, Lisbon, Portugal; ^17^LIP/PC2S Lab, Université Grenoble Alpes, Grenoble, France; ^18^Department of Biomedicine, Biotechnology and Public Health, University of Cadiz, Cadiz, Spain; ^19^Instituto ACT, Madrid, Spain; ^20^Department of Psychology, European University of Madrid, Madrid, Spain; ^21^Department of Psychology and Sociology, University of Zaragoza, Zaragoza, Spain; ^22^Department of Mental Health, Heim Pal National Pediatric Institute, Budapest, Hungary; ^23^Institute of Behavioural Sciences, Semmelweis University, Budapest, Hungary; ^24^Bartosz Kleszcz Psychotherapy and Training, Sosnowiec, Poland; ^25^Department of Psychology, University of Jyväskylä, Jyväskylä, Finland; ^26^Department of Child Psychiatry, Institute for Children’s Diseases, Clinical Centre of Montenegro, Podgorica, Montenegro; ^27^Department of Child, Adolescent and Developmental Neurology, University Children’s Hospital, University Medical Center, Ljubljana, Slovenia; ^28^Département de Psychologie, Université du Québec à Trois-Rivières, Trois-Rivières, QC, Canada; ^29^Department of Psychiatry and Behavioral Science, Duke University, Durham, CA, United States; ^30^Department of Psychology, University of Cyprus, Nicosia, Cyprus

**Keywords:** prosocial behavior, well-being, COVID-19 pandemic, predictors of prosocial behavior, social support

## Abstract

The coronavirus disease (COVID-19) pandemic fundamentally disrupted humans’ social life and behavior. Public health measures may have inadvertently impacted how people care for each other. This study investigated prosocial behavior, its association well-being, and predictors of prosocial behavior during the first COVID-19 pandemic lockdown and sought to understand whether region-specific differences exist. Participants (*N* = 9,496) from eight regions clustering multiple countries around the world responded to a cross-sectional online-survey investigating the psychological consequences of the first upsurge of lockdowns in spring 2020. Prosocial behavior was reported to occur frequently. Multiple regression analyses showed that prosocial behavior was associated with better well-being consistently across regions. With regard to predictors of prosocial behavior, high levels of perceived social support were most strongly associated with prosocial behavior, followed by high levels of perceived stress, positive affect and psychological flexibility. Sociodemographic and psychosocial predictors of prosocial behavior were similar across regions.

## Introduction

The outbreak of the coronavirus disease (COVID-19) in late 2019 quickly led nations to declare states of emergency due to the overwhelming demands posed by the rapid spread of the virus globally. As a response to the pandemic, a range of behavioral measures were implemented, which drastically impacted people’s everyday life and functioning. In an attempt to curtail COVID-19 transmissions, many governments around the world declared national lockdowns, enforced curfews and travel bans, temporarily closed schools, businesses, cultural and recreational facilities, and issued “stay-at-home” recommendations or orders (World Health Organization [WHO], 2020). These physical distancing and quarantine policies severely disrupted daily social interactions and resulted in increased social isolation (Jarvis et al., 2020; Smith and Lim, 2020), a situation at odds with basic human needs to connect with others (Baumeister and Leary, 1995). The absence of social interactions along with emerging feelings of fear, insecurity, and stress (Luo et al., 2020; Wang et al., 2020) have limited the possibilities to assist others and may give rise to self-centered concern and disregard of others. Indeed, a variety of reactions characterized as selfish or antisocial were observed as a result of the pandemic and related measures. These included non-compliance with public health measures (e.g., mask-wearing), which increases risk of transmission, and issues such as overbuying and food stockpiling (Dholakia, 2020; Wang et al., 2020), and even a sharp rise of reports and acts of racism and xenophobia against Asian people (Ng, 2020), when the coronavirus spread in spring 2020. Moreover, recent research points to the importance of social norms when individuals inform their decisions about behaving more prosocially or in in a self-interested way (Abel and Brown, 2020). Being exposed to positive role models might be curtailed due to a limited participation in social life.

However, this unique test of our social fabric simultaneously revealed prosocial responses, which encompass a variety of attitudes and voluntary actions that may be adopted by individuals, all aiming at helping, supporting, comforting, or caring for others (Batson and Powell, 2003). During the first wave of the pandemic in 2020, solidarity and cooperation were reflected in various individual behaviors and collective efforts. For example, measures were adhered to by large parts of the population in order to protect the public health (Margraf et al., 2021), as reflected by a rapid surge of millions of people across the globe practicing scientifically recommended hygiene measures and self-isolating. Cooperation was further manifested in musicians performing concerts from their balconies for the common good and people applauding from their windows to express gratitude to frontline and healthcare workers (Taylor, 2020). Collective efforts to directly help those in need further entailed the establishment of formal volunteering endeavors and informal initiatives to support neighbors in communities (Beardmore et al., 2020). Indeed, previous research has described the emergence of prosocial responses to shared experiences of adversity and suffering (Staub, 2003, 2005). According to this perspective, experiencing trauma generates a sense of shared fate and identity, which may lead to increased empathy with and a greater motivation to help those in need. Importantly, these outbursts of altruistic and prosocial behaviors have been observed in the various contexts of traumatic life events, such as interpersonal conflict, war, and natural disasters (Kaniasty and Norris, 1995; Hartman and Morse, 2020). It has been suggested that in the context of such extreme and severe disasters, populous acts of cooperation and prosocial behavior are likely facilitated by a higher number of opportunities to help others (Vollhardt, 2009). The COVID-19 crisis presented an emergency situation in which different helping behaviors were difficult to accomplish. The governmentally mandated lockdowns in the wake of the pandemic thus provided a unique context to study other-oriented behavior. Little is known about how periods of increased insecurity, stress, and social disruption – as experienced during the first COVID-19 lockdowns – are related to individuals’ readiness and opportunities to demonstrate prosocial behavior.

Engaging in prosocial behavior may be a potent protective factor during periods of global adversity, as it can be linked to benefits on the individual and to society as a whole: Acts that focus on benefitting others are particularly important in the face of widespread suffering caused by the COVID-19 pandemic, in which many individuals were confronted with financial hardship, social isolation, and the loss of loved ones. Furthermore, prosocial behavior is associated with personal benefits for the individual provider of help. This is supported by mounting evidence of different types of prosocial behavior being related to greater physical and mental health with studies showing that volunteering, family caregiving, and the provision of tangible help to close others are associated with greater longevity (Harris and Thoresen, 2005; Poulin et al., 2013; Roth et al., 2013) and that spending money on others augments emotional well-being (Aknin et al., 2013). Past research has shown that prosocial behavior buffers against the negative effects of daily stress on emotional well-being (Raposa et al., 2016). Furthermore, it was found that time spent volunteering after a natural disaster is associated with increased feelings of belongingness (Gordon et al., 2011). In the context of the current global crisis, a recent experimental study in a United States sample provides preliminary evidence that showing prosocial behavior during the COVID-19 pandemic increases positive affect, empathy, and social connectedness (Varma et al., 2020), indicating that helping others has an immediate impact on the helper’s mood.

Prosocial behavior is in part driven by feelings of empathy and a concern for the welfare of others (Eisenberg and Miller, 1987; Carlo et al., 1996). However, most previous research with regard to predictors of prosocial behavior has focused on individual emotional competencies, leaving out the situational and social context. Similarly, the majority of studies that examine predictors of prosocial behavior have been conducted during non-emergency situations. To the extent that individuals can show varied emotional and behavioral responses to the COVID-19 crisis, the factors influencing prosocial behavior may differ in terms of situational contexts, particularly those with an increased global burden and in times of (mandated) social distancing. Understanding prosocial responding during the COVID-19 pandemic therefore necessitates an examination of psychosocial variables that likely play an important role with regard to prosocial behavior.

### Perceived Social Support

The availability of supportive relationships has been linked to increased levels of prosocial behavior. The social network provides an essential context in which prosocial behavior can be displayed. Moreover, conceptual links between emotional functioning and prosocial behavior have been suggested in that experiencing social support can foster social-emotional competencies (Eisenberg and Fabes, 1990), such as providing help and support to others. In addition, the experience of social exclusion has been shown to be associated with decreases in prosocial acts (Twenge et al., 2007). While social interactions have been compromised drastically during the first wave of COVID-19 lockdowns, the perception of social support or a lack thereof in a novel, encumbering situation might be sharpened. We therefore hypothesized that a high level of perceived social support would predict more prosocial behavior.

### Psychological Flexibility

Psychological flexibility has been proposed as a cornerstone of health and well-being (Kashdan and Rottenberg, 2010; Leonidou et al., 2019). Psychological flexibility comprises a range of intra- and interpersonal skills enabling an individual to shift their mindsets and adapt their behavioral repertoire to what a situation affords. It has been shown to protect from the negative effects of daily stress (Gloster et al., 2017) and major life events (Fonseca et al., 2020) on well-being and depressive symptoms. Research on psychological flexibility postulates that responding flexibly to situational demands allows individuals to prioritize areas in life that are meaningful and consistent with personal values (Hayes et al., 2006; Villanueva et al., 2020). Additionally, researchers started to evaluate psychological flexibility as a potential public health target with preliminary evidence of its potency: In a recent study, it was found that a brief intervention strengthening psychological flexibility led to an increase in prosocial choices in couples (Gloster et al., 2020c). Another recent study from our research group has supported the dual roles of psychological flexibility and prosocial behaviors in mitigating the impact of illness perception toward COVID-19 on mental health in a sample of Hong Kong adults during the early phase of the pandemic (Chong et al., 2021). Given that the COVID-19 lockdown measures may make it harder for individuals to shape their daily lives in accordance with their personally held values, we hypothesized that high levels of psychological flexibility would be positively associated with the occurrence of prosocial behavior.

### Perceived Stress

A growing body of research supports the hypothesis that experiencing stress elicits prosocial behavior (Buchanan and Preston, 2014). Proponents of this perspective postulate that affiliative behaviors present a way of coping with the adverse experiences of stress (Midlarsky, 1991; Taylor et al., 2000). This view is supported by empirical data showing an increase in prosocial behavior under stress in some situations. For example, studies have shown that people exhibit prosocial behavior under time pressure (Rand et al., 2012). In an experimental study, the experience of an acute stressor led to increases in trustworthiness and prosocial behavior in social interactions (von Dawans et al., 2012), suggesting that feeling stressed contributes to the emergence of prosocial behaviors due to its stress-buffering properties. Alternatively, engaging in activities such as volunteering, helping, and supporting others require personal resources, which may increase stress (Gloster et al., 2020a). Within the pandemic, prosocial behaviors might be one way to overcome experiences of stress that result from being affected or by observing other peoples’ suffering during the pandemic. We therefore hypothesized that high levels of perceived stress would be positively related to prosocial behavior.

### Positive Affect

A vast amount of evidence supports the association between positive mood and prosocial behavior. One prevailing theoretical assumption explaining this relationship is that prosocial behavior is a strategy to maintain positive mood. Experimental evidence shows that the induction of positive mood facilitates helping behavior (Rosenhan et al., 1981) and that positive state affect leads to increased prosocial behavior in the work context (George, 1991). Support of this association is provided by numerous studies manipulating positive affect in different ways, which show, for example, that prosocial behavior occurs following the reception of a surprise payment (Isen and Levin, 1972) or after listening to happy thoughts (O’Malley and Andrews, 1983). We therefore hypothesized that positive affect would contribute to the emergence of prosocial behavior during this time of elevated stress.

### The Present Study

The positive impact of prosocial behavior on happiness has been documented in Western and non-Western societies and across socioeconomic status (Dunn et al., 2008; Aknin et al., 2013). However, it remains unexplored whether this association exists during a long-lasting incisive event limiting humans’ social behavior faced by almost the entire world population concurrently. The current study aimed to extend our understanding of prosocial behavior in the face of an adverse global situation, which disrupts social life and everyday social interactions. First, the present study examined whether and to which extent people engage in prosocial behaviors during the first COVID-19 pandemic lockdowns. Second, this study investigated the relationship between prosocial behavior and well-being during the first wave of COVID-19 pandemic lockdowns. Third, we aimed at identifying predictors of prosocial behavior during the COVID-19 pandemic lockdowns by testing the relationships between sociodemographic and psychosocial variables and prosocial behavior. Lastly, we were interested in investigating region-specific differences in the extent of prosocial behavior, its association with well-being, and predictors of prosocial behavior.

## Materials and Methods

### Procedure

The current study was part of the COVID-Impact project, an international cross-sectional online survey conducted in 78 countries worldwide (Gloster et al., 2020b). The aim of the COVID-Impact project was to explore the behavioral, emotional, and psychological response to the COVID-19 pandemic and related lockdown measures. Between April and June 2020, data were collected through social media, university mailing lists, and advertisements in professional associations using a secured google platform. Participants were first informed about the study aim, the procedure, and about risks and benefits of taking part. Those who chose to participate gave informed consent electronically. The survey was available in 22 different languages. Ethical approval was granted by the Cyprus National Bioethics Committee (ref.: EEBK EΠ 2020.01.60 on 3rd April 2020) and by the local ethics boards from the research teams involved in data collection.

### Measures

#### Well-Being

The Mental Health Continuum-Short Form (MHCS-SF) was employed, which is a widely used and validated measure for positive mental health (Lamers et al., 2011). The instrument comprises 14 items describing various feelings, which are rated on a 6-point Likert scale relating to the frequency of occurrence (0 = “never” to 6 = “every day”). The MHC-SF contains three subscales with three items measuring emotional, six items measuring psychological, and five items measuring social well-being. The scale revealed a Cronbach’s alpha of 0.91 in this sample. The total score is comprised of the sum score of all items (ranging from 0 to 70).

#### Prosocial Behavior

Prosocial behavior was measured using six out of 16 items of the Prosocialness Scale for Adults (PSA) (Caprara et al., 2005). We were not able to include all items of the scale in order to avoid burdening respondents. The six items refer to statements about helping and sharing with friends and others, being available for volunteer activities, being empathetic with those in need, and spending time with lonely people. Participants were asked to rate the frequency of occurrence of the stated behavior on a 5-point Likert scale (1 = “never/almost never” to 5 = “always/almost always”). We used a sum score of the six items (range: 6–36), with higher values indicating more prosocial behavior. In this sample, Cronbach’s alpha was 0.83 for the scale.

#### Sociodemographic Status

Sociodemographic predictors included age, gender, education level, employment status, marital status, and living situation.

#### Characteristics Related to Quarantine/Self-Isolation

Participants responded to questions related to the amount of time in lockdown (in weeks) and the impact of the lockdown on their financial situation (“have got better,” “stayed the same,” “have gotten worse”) and on daily activities (“did not leave house,” “left house once only,” “left house a couple of times,” “left house more than three times per week”).

#### Social Support

The Oslo 3-item Social Support Scale (OSSS-3) was used to measure the availability of social support by asking about the number of close people, the extent of concern and interest, and the appraised ease of getting help from neighbors (Dalgard et al., 2006). Analysis of the internal consistency revealed Cronbach’s alpha of 0.54. The sum score is classified in groups of different levels of social support: low (3–8), moderate (9–11), and high (12–14).

#### Psychological Flexibility

Psy-Flex is a 6-item instrument that measures psychological flexibility, a construct referring to a range of intra- and interindividual skills that allow an open and presently aware mindset as well as the clarification and pursuit of deeply held, personal values (Gloster et al., 2021). Items were rated on a 5-point Likert scale related to the frequency of occurrence (1 = “very seldom” to 5 = “very often”). We used a sum score of all items. The internal consistency of the scale revealed a Cronbach’s alpha of 0.84 in this sample.

#### Perceived Stress

The 10-item Perceived Stress Scale (PSS) was used to measure the degree to which life situations of the past week are appraised as stressful (Cohen et al., 1983). Respondents were asked to rate the frequency of feeling or thinking about life situations or events in a certain manner on a 5-point Likert scale (0 = “never” to 4 = “very often”). The sum score of all items was used in this sample with internal consistency (α = 0.89). The scores are classified into groups of low (0–13), moderate (14–26), and high stress (27–40).

#### Positive Affect

The subscale of the Positive and Negative Affect Scale (PANAS; Watson et al., 1988) was used to measure positive affect. The subscale is comprised of 10 items that are scored on a 5-point Likert scale (1 = “very slightly/little” to 5 = “extremely”). Sum score of all items was calculated; internal consistency analysis revealed a Cronbach’s alpha of 0.90.

### Statistical Analysis

Descriptive statistics included relative frequencies, means and standard deviations (SDs), or medians and interquartile ranges (IQR) of sociodemographic variables (age, gender, education level, employment status, marital status, children, and living situation), characteristics regarding the self-isolation/quarantine measures (weeks in quarantine, having been infected by COVID-19, impact of social isolation on financial situation and daily activities), and predictor and outcome variables for the overall sample and for all regions. Countries were grouped into eight geo-cultural regions (Southern Europe (=SE) includes Cyprus, Greece, Spain, Italy, Portugal, and Andorra; Eastern Europe (=EE) includes Latvia, Poland, Czech Republic, Hungary, Slovakia, Slovenia, Croatia, Ukraine, Romania, Serbia, Montenegro, and North Macedonia; Western Europe (=WE) includes Austria, Switzerland, Liechtenstein, Germany, Belgium, Netherlands, Luxembourg, France, United Kingdom, and Ireland; Northern Europe (=NE) includes Finland, Denmark, Sweden, Norway, and Iceland; Western Asia (=WA) includes Turkey, Azerbaijan, Lebanon, Israel, Jordan, Iran, Pakistan, Kuwait, Saudi-Arabia, and United Arab Emirates; and East Asia (=EA) includes Hong Kong, China, and Taiwan; Latin America (=LA) includes Mexico, El Salvador, Guadeloupe, Panama, Colombia, Ecuador, Brazil, Peru, Uruguay, Paraguay, Argentina, and Chile; North America (=NA) includes Canada, United States. In order to analyze regional variations of prosocial behavior, we compared the regions’ mean to the mean of the overall sample with a linear regression model for prosocial behavior centering around the grand mean and region as a predictor. Cohen’s *d* for the standardized difference was used to measure the magnitude of the effect with values. Bivariate correlation analysis (*r*) for the entire sample was used to assess associations between all study variables (predictors and outcome) with ≤0.10 referring to very small, ≤0.20 to small, ≤0.30 to moderate, ≤0.40 to large and >0.40 to very large effect sizes (Funder and Ozer, 2019).

First, simple linear regressions were performed with prosocial behavior as predictor and well-being as outcome in the total sample and in each region’s subsample. Next, separate multiple regression analyses with the total sample and with the region-samples were performed with prosocial behavior as the dependent variable and each set of predictors (sociodemographic and psychosocial variables) as the independent variables. Standardized regression coefficients (Beta) with 95% Confidence Intervals (CI) were computed as indices of effect size in order to measure the strength of the association between each predictor and outcome variable. Variance Inflation Factors (VIF) were used to check for multicollinearity between the predictors. All analyses were first conducted with the overall sample and subsequently with the subsamples of each region. All analyses were computed using R software version 1.3.959 (R Core Team).

## Results

The sample comprised *N* = 9,496 participants from 60 countries grouped into eight regions: Southern Europe (*n* = 2,820), Eastern Europe (*n* = 2,269), Western Europe (*n* = 2,107), Northern Europe (*n* = 172), West Asia (*n* = 720), and East Asia (*n* = 520), Latin America (*n* = 560), and North America (*n* = 328). Regions with *n* < 100 were not considered in the sample. Table 1 presents relative frequencies and, where appropriate, measures of central tendency of the sociodemographic variables and characteristics related to quarantine/self-isolation for the entire sample and individual regions.

**TABLE 1:** Sociodemographic and characteristics related to quarantine/self-isolation of the total sample and subsamples of each region.

		All	SE	EE	WE	NE	WA	EA	LA	NA
		*N* = 9,496	*n* = 2,820	*n* = 2,269	*n* = 2,107	*n* = 172	*n* = 720	*n* = 520	*n* = 560	*n* = 328
**Sociodemographic characteristics**										
Sex, %	Female	77.6	77.3	81.1	76.9	82.0	68.8	73.7	78.4	83.2
	Male	22.0	22.4	18.8	22.5	16.9	30.9	26.0	21.4	15.5
	Other	0.4	0.2	0.1	0.6	1.2	0.3	0.4	0.2	1.2
Age, mean (SD)		37.2 (13.3)	37.2 (13.3)	37.7 (12.9)	39.1 (15.9)	35.9 (14.0)	30.8 (11.1)	32.7 (11.5)	34.5 (13.5)	39.6 (15.9)
Education, %	Primary school	0.8	0.5	1.3	0.4	0.6	2.1	0.0	0.7	1.5
	High school	11.7	9.9	14.9	5.9	22.1	28.3	8.5	10.9	6.1
	College/University	12.9	15.6	9.7	14.6	16.3	4.9	9.4	17.5	15.9
	Graduated from College/University	29.0	28.1	30.8	18.5	26.2	45.8	46.5	29.5	26.8
	Master/Postgraduate	34.4	36.2	35.4	42.1	22.1	11.1	28.8	34.6	27.7
	Doctoral level	8.6	7.2	6.7	13.5	7.6	6.0	6.5	3.8	20.7
	Other	2.6	2.6	1.2	5.0	5.2	1.8	0.2	3.0	1.2
Employment, %	Working (full-time)	53.7	53.8	64.2	46.9	49.4	42.9	63.1	45.9	51.2
	Working (part-time)	17.6	16.4	12.2	30.1	18.6	6.4	14.6	17.3	14.6
	Unemployed	22.7	23.5	17.1	17.0	25.0	47.2	19.8	32.5	22.3
	On parental leave	2.3	2.6	3.4	1.8	1.7	1.2	0.4	0.9	2.4
	Retired	3.7	3.7	3.2	4.2	5.2	2.2	2.1	3.4	9.5
Marital status, %	Single	30.8	35.0	24.9	27.5	24.7	44.8	45.9	44.5	26.2
	Married	36.1	40.3	39.3	36.3	28.3	36.9	31.8	31.5	48.2
	Relationship/engaged	25.7	22.6	31.5	33.8	45.8	17.3	21.9	21.5	23.0
	Divorced	5.1	0.0	0.0	0.0	0.0	0.0	0.0	0.0	0.0
	Widowed	1.1	0.8	2.5	0.8	1.2	0.3	0.2	0.8	1.6
Children, %	Yes	40.7	40.7	47.4	42.3	33.1	31.8	23.3	37.8	41.8
	No	59.3	59.3	52.6	57.7	66.9	68.2	76.7	62.7	58.2
Living situation, %	Alone	14.6	14.4	17.5	19.3	32.3	8.9	7.8	9.3	15.9
	With parents	20.8	24.3	13.7	10.4	6.0	54.5	47.8	30.5	10.6
	With one parent	5.1	4.9	5.6	3.5	0.0	4.5	7.0	15.3	3.6
	With own family	54.1	56.4	63.1	66.8	61.7	32.3	37.3	44.9	69.9
	With friends/roommates	5.5	0.0	0.0	0.0	0.0	0.0	0.0	0.0	0.0
**Characteristics related to lockdown/self-isolation**										
Weeks in lockdown/self-isolation, median (IQR)		5 (3)	6 (3)	5 (3)	6 (3)	6 (4)	4 (2)	12 (8)	5 (3)	5 (2)
Infected by COVID-19, %	Yes	1.4	0.7	0.7	0.7	10.5	8.1	0.2	0.0	2.4
	No	88.0	92.8	85.4	83.5	70.3	86.2	98.5	95.4	78.4
	Symptoms, unsure	10.6	6.5	14.0	15.8	19.2	5.7	1.3	4.6	19.2
Changes in financial situation, %	Has gotten better	8.9	9.4	6.6	12.9	5.8	6.7	7.5	5.9	8.8
	Stayed the same	57.9	51.5	64.0	59.5	73.8	59.4	62.7	43.8	63.1
	Has gotten worse	33.3	39.1	29.4	27.6	20.4	33.9	29.8	50.4	28.0
Left house since isolation, %	No	47.7	52.7	44.5	33.8	55.2	63.7	33.8	70.7	59.8
	Once only	7.6	8.2	7.5	5.4	9.9	11.7	9.4	5.9	7.9
	A couple of times	23.7	23.4	22.8	30.8	13.4	14.0	29.0	16.4	18.3
	>three times per week	20.9	15.7	25.2	30.0	21.5	10.6	27.7	7.0	14.0

*Countries were clustered in regions as follows: Southern Europe (=SE) includes Cyprus, Greece, Spain, Italy, Portugal, and Andorra; Eastern Europe (=EE) includes Latvia, Poland, Czech Republic, Hungary, Slovakia, Slovenia, Croatia, Ukraine, Romania, Serbia, Montenegro, and North Macedonia; Western Europe (=WE) includes Austria, Switzerland, Liechtenstein, Germany, Belgium, Netherlands, Luxembourg, France, United Kingdom, and Ireland; Northern Europe (=NE) includes Finland, Denmark, Sweden, Norway, and Iceland; Western Asia (=WA) includes Turkey, Azerbaijan, Lebanon, Israel, Jordan, Iran, Pakistan, Kuwait, Saudi-Arabia, and United Arab Emirates; and East Asia (=EA) includes Hong Kong, China, Taiwan; Latin America (=LA) includes Mexico, El Salvador, Guadeloupe, Panama, Colombia, Ecuador, Brazil, Peru, Uruguay, Paraguay, Argentina, and Chile; North America (=NA) includes Canada, United States.*

### Frequency and Types of Prosocial Behavior

Overall, prosocial behavior was reported to occur often on average in the total sample (*M* = 22.8, *SD* = 4.2). Different types of prosocial behaviors were reported with similar frequency on average. On a descriptive level, lowest levels were reported for being available for volunteering activities (*M* = 3.2, *SD* = 1.2) and spending time with friends who feel lonely (*M* = 3.4, *SD* = 1.0), higher levels for sharing with friends (*M* = 3.9, *SD* = 0.92), willing to help (*M* = 4.1, *SD* = 0.8), trying to help others (*M* = 4.1, *SD* = 0.8), and being empathetic with those in need (*M* = 4.1, *SD* = 0.8). With regard to regional variations, the reported levels of prosocial behavior were largely similar across regions. However, two regions reported slightly higher levels of prosocial behavior (Southern Europe, medium effect; Western Asia, large effect), and other two regions demonstrated lower levels of prosocial behavior (Eastern Europe, medium effect; Eastern Asia, very large effect) than the average sample. Average levels of prosocial behavior and differences between regions and their effect sizes can be found in Table 2.

**TABLE 2:** Average levels of prosocial behavior across regions.

	*M (SD)*	Difference between country mean and overall mean (95% CI)	Effect size
**Regions**			
Total sample	22.8 (4.2)		
Southern Europe	24.0 (3.8)	1.27 (1.02, 1.35)	**0.295**
Eastern Europe	21.0 (4.3)	−1.74 (−2.03, −1.64)	**0.433**
Western Europe	23.2 (3.8)	0.45 (0.17, 0.53)	0.088
Northern Europe	22.4 (3.8)	−0.31 (−0.99, 0.17)	0.102
West Asia	24.1 (3.9)	1.38 (0.99, 1.59)	**0.319**
East Asia	20.7 (4.0)	−2.06 (−2.51, −1.80)	**0.529**
Latin America	23.2 (4.3)	0.46 (−0.00, 0.74)	0.087
North America	23.3 (3.9)	0.55 (0.03, 0.89)	0.114

*M, mean; SD, standard deviation; CI, confidence interval, Cohen’s d for standardized difference between region mean and overall mean; moderate and large effects are printed in bold.*

Regional variations were observed with regard to specific types of prosocial behavior. In most regions, about 45–55% of the respondents indicated being available for volunteer activities as “often” or “almost always”, whereas this was the case for only 20.8% in Eastern Europe, 27.3% in Northern Europe, and 26.1% in East Asia. A similar pattern emerged on spending time with friends who feel lonely: In most regions, between 47 and 62% indicated that this type of behavior occurred as “often” or “almost always”, while this was true for 34% in Eastern Europe, 37.8% in Northern Europe, and 36% in East Asia.

### Relationship Between Prosocial Behavior and Well-Being

Scatterplot and Pearson’s correlation showed a moderate association between prosocial behavior and well-being (*r* = 0.32). Simple linear regression revealed that prosocial behavior explained a significant amount of variance in well-being, *F*(1,9483) = 1,096, *p* < 0.001, *R*^2^_adjusted_ = 0.104. The regression coefficient [*B* = 1.07, CI (1.01, 1.13)] indicates that well-being increases by 1.07 for each unit of increase in prosocial behavior [β = 0.32, CI (0.30, 0.34), *p* < 0.001].

**FIGURE 1:**
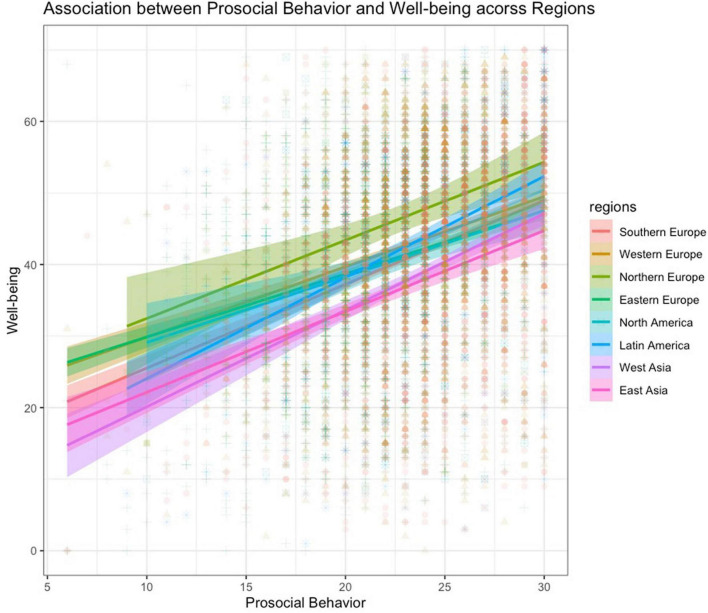
Association between prosocial behavior and well-being across regions.

With regard to regional variations, prosocial behavior significantly positively predicted well-being in all regions with the largest effect in Latin America [β = 0.41, CI (0.29, 0.36), *p* < 0.001], West Asia [β = 0.38, CI (0.31, 0.45), *p* < 0.001] and East Asia [β = 0.35, CI (0.27, 0.44), *p* < 0.001] followed by Southern Europe [β = 0.32, CI (0.29, 0.36), *p* < 0.001], Northern Europe [β = 0.32, CI (0.23, 0.32), *p* < 0.001], Eastern Europe [β = 0.27, CI (0.24, 0.32), *p* < 0.001] and Western Europe [β = 0.27, CI (0.23, 0.31), *p* < 0.001], and North America [β = 0.24, CI (0.14, 0.35), *p* < 0.001]. Figure 1 presents the association between prosocial behavior and well-being across regions.

### Predictors of Prosocial Behavior

Descriptive statistics of all psychosocial variables can be found in Table 3, displaying means and SDs for the outcome and predictor variables for the total sample and subsamples of each region.

**TABLE 3:** Description of predictors of the total sample and subsamples of each region.

	All	SE	EE	WE	NE	WA	EA	LA	NA
	*N* = 9,496	*n* = 2,820	*n* = 2,269	*n* = 2,107	*n* = 172	*n* = 720	*n* = 520	*n* = 560	*n* = 328
**Predictors**									
*Social Support, M (SD)*	9.9 (2.1)	10.1 (2.1)	9.7 (2.0)	10.5 (2.1)	9.7 (1.9)	9.9 (2.2)	8.2 (2.0)	9.5 (2.2)	9.8 (2.4)
*Low %*	24.3	20.8	26.2	15.3	25.6	28.1	56.0	31.2	28.4
*Moderate, %*	52.4	53.5	56.2	52.1	55.8	47.4	39.6	51.8	48.2
*High, %*	23.3	25.7	17.5	32.6	18.6	24.6	4.4	17.0	23.5
*Perceived Stress, M (SD)*	17.1 (7.5)	16.5 (7.2)	17.3 (8.0)	16.2 (7.2)	16.3 (6.9)	19.5 (6.6)	19.9 (6.2)	16.2 (8.3)	18.1 (7.7)
*Low %*	33.1	34.8	33.2	38.2	37.8	19.2	14.2	40.7	29.9
*Moderate, %*	55.8	55.9	53.2	53.3	53.5	66.5	72.9	48.2	54.3
*High, %*	11.1	9.3	13.6	8.5	8.7	14.3	12.9	11.1	15.9
*Psych. Flexibility, M (SD)*	21.8 (4.1)	22.0 (3.9)	22.1 (3.9)	22.6 (4.1)	22.6 (3.9)	20.2 (4.0)	19.5 (4.0)	21.2 (4.5)	22.1 (4.7)
*Positive Affect, M (SD)*	29.0 (8.1)	30.1 (8.1)	28.1 (7.7)	29.3 (7.9)	26.7 (5.9)	30.4 (8.2)	25.7 (7.2)	30.2 (8.8)	25.1 (8.3)

*Countries were clustered in regions as follows: Southern Europe (=SE) includes Cyprus, Greece, Spain, Italy, Portugal, and Andorra; Eastern Europe (=EE) includes Latvia, Poland, Czech Republic, Hungary, Slovakia, Slovenia, Croatia, Ukraine, Romania, Serbia, Montenegro, and North Macedonia; Western Europe (=WE) includes Austria, Switzerland, Liechtenstein, Germany, Belgium, Netherlands, Luxembourg, France, United Kingdom, and Ireland; Northern Europe (=NE) includes Finland, Denmark, Sweden, Norway, and Iceland; Western Asia (=WA) includes Turkey, Azerbaijan, Lebanon, Israel, Jordan, Iran, Pakistan, Kuwait, Saudi-Arabia, and United Arab Emirates; and East Asia (=EA) includes Hong Kong, China, Taiwan; Latin America (=LA) includes Mexico, El Salvador, Guadeloupe, Panama, Colombia, Ecuador, Brazil, Peru, Uruguay, Paraguay, Argentina, and Chile; North America (=NA) includes Canada, United States.*

Table 4 presents means and SDs, as well as bivariate correlations among all psychological variables – predictors and outcome(s) – across the entire sample. Well-being showed a strong positive correlation with psychological flexibility, and positive affect, and a moderate to strong negative association with perceived stress. Prosocial behavior showed moderate positive correlations with well-being, social support, and positive affect; a weak positive correlation with psychological flexibility; and a weak negative correlation with perceived stress.

**TABLE 4:** Correlation matrix for outcome and predictor variables.

Variable (No.)	Mean (SD)	Range	1	2	3	4	5	6
Prosocial behavior (1)	22.8 (4.18)	(6–30)	1.00	**0.32**	**0.34**	−0.09	0.21	**0.30**
Well-being (2)	48.34 (11.38)	(0–70)		1.00	**0.45**	−**0.54**	**0.54**	**0.64**
Social Support (3)	9.9 (2.1)	(3–14)			1.00	−0.26	**0.32**	**0.34**
Perceived Stress (4)	17.1 (7.5)	(0–40)				1.00	−**0.52**	−**0.49**
Psych. Flexibility (5)	21.8 (4.1)	(6–30)					1.00	**0.50**
Positive Affect (6)	28.07 (4.6)	(10–50)						1.00

*Higher values indicate greater extent of the measured trait; strong correlations are printed in bold; all correlations are statistically significant, p < .001, two-tailed.*

Table 5 displays the results of the multiple regression analysis for the overall sample [*F*(24, 9407) = 88.92, *p* < 0.001, *R*^2^_adjusted_ = 0.183]. Both sociodemographic and psychosocial predictors were significant, with several psychosocial predictors showing the largest effects on prosocial behavior in the overall sample. Arranged in order of predictor strength, a high level of perceived social support was the predictor that best explained prosocial behavior (relative to low levels of social support). Higher levels of perceived stress and positive affect also contributed significantly to variation in prosocial behavior. This was followed by female gender (relative to male), being retired (relative to working full time), and living with friends or roommates (relative to living alone) as positive predictors. Significant positive predictors of prosocial behavior were higher levels of psychological flexibility, being retired, being unemployed (relative to working full time), living with parents, and living with roommates (relative to living alone). Significant negative predictors of prosocial behavior were living with own family (relative to living alone), as well as being in a relationship and being widowed (relative to being married).

**TABLE 5:** Sociodemographic and psychosocial predictors of prosocial behavior.

		Total sample			
Sociodemographic predictor		*B*	*SE B*	β	(95% CI)
Age		−0.02**	0.00	0.00**	(−0.00, −0.00)
Gender	Male	Ref		Ref	
	Female	1.04**	0.10	0.25**	(0.21 −0.31)
Employment	Working (full time)	Ref		Ref	
	Working (part time)	0.05	0.11	0.01	(−0.04, 0.06)
	Unemployed	0.37**	0.12	0.09**	(0.04, 0.14)
	On parental leave	−0.42	0.27	−0.10	(−0.23, 0.02)
	Retired	0.50*	0.23	0.12*	(0.01, 0.23)
Living situation	Alone	Ref		Ref	
	With parents	0.40**	0.15	0.10**	(0.03, 0.17)
	With one parent	0.08	0.21	0.02	(−0.08, 0.12)
	With own family	−0.48**	0.15	−0.12**	(−0.19, −0.05)
	With friends/roommates	0.47*	0.20	0.11*	(0.02, 0.21)
Marital status	Married	Ref		Ref	
	In a relationship	−0.29*	0.12	−0.07*	(−0.13, −0.01)
	Single	−0.01	0.15	0.00	(−0.07, 0.07)
	Divorced	−0.23	0.20	−0.05	(−0.50, −0.11)
	Widowed	−1.21**	0.38	−0.29**	(−0.34, −0.01)
Changes in finances	Got better	Ref		Ref	
	Same	−0.14	0.14	−0.03	(−0.10, 0.03)
	Got worse	0.25	0.15	0.06	(−0.01, 0.13)
**Psychosocial predictors**					
Perceived social support	Low	Ref		Ref	
	Moderate	1.44**	0.10	0.34**	(0.30, 0.40)
	High	2.71**	0.12	0.65**	(0.59, 0.70)
Perceived stress	Low	Ref		Ref	
	Moderate	0.27**	0.09	0.07**	(0.02, 0.11)
	High	1.20**	0.16	0.29**	(0.29, 0.04)
Psychological flexibility		0.10**	0.01	0.10**	(0.07, 0.12)
Positive affect		0.13**	0.01	0.25**	(0.23, 0.27)

R^2^ = 0.18 (p < 0.001); β, standardized regression coefficient; CI, confidence interval; *p < 0.05; **p < 0.001.

Table 6 demonstrates a simplified version of the multiple regression analyses for each region. With regard to regional variation, the multiple regression analyses revealed the following results: The strongest predictors of prosocial behavior were high levels of perceived social support in all regions with East Asia [β = 0.84, CI (0.44, 1.23), *p* < 0.001], West Asia [β = 0.75, CI (0.55,0.95), *p* < 0.001], Northern Europe [β = 0.72, CI (0.24, 1.20), *p* < 0.001], Eastern Europe [β = 0.67, CI (0.55,0.80), *p* < 0.001], Northern America [β = 0.64, CI (0.33, 0.95), *p* < 0.001], Latin America [β = 0.58, CI (0.33, 0.82), *p* < 0.001], Western Europe [β = 0.57, CI (0.43, 0.70), *p* < 0.001] and Southern Europe [β = 0.53, CI (0.43, 0.64), *p* < 0.001].

**TABLE 6:** Simplified representation of significant predictors of prosocial behavior for each region.

		SE	EE	WE	NE	WA	EA	LA	NA
**Sociodemographic predictor**									
Age		−X	−X				−X		−X
Gender	Female	X	X	X	X	X	X	X	X
Employment	Working (part time)								
	Unemployed				X	−X	X		
	On parental leave								
	Retired								X
Living situation	With parents		X						
	With one parent								X
	With own family	X	−X					−X	
	With friends or roommates	−X		X					
Marital status	In a relationship							−X	
	Single		−X					−X	
	Divorced				−X				
	Widowed								
Changes in finances	Same								
	Got worse		X			X			X
**Psychosocial predictors**									
Perceived social support	Moderate	X	X	X	X	X	X	X	X
	High	X	X	X	X	X	X	X	X
Perceived stress	Moderate		X						
	High	X	X	X			X		X
Psychological flexibility		X		X				X	
Positive affect		X	X	X		X	X	X	X
Overall adjusted *R*^2^ (%)		17.9	16.8	15.7	12.6	19.2	22.5	19.4	21.9

*Southern Europe (SE): n = 2,820; Western Europe (WE): n = 2,107; Northern Europe (NE): n = 172; Eastern Europe (EE): n = 2,269; North America (NA): n = 328; Latin America (LA): n = 560; Western Asia (WA): n = 720; Eastern Asia (EA): n = 520; X = significant positive predictor, −X = significant negative predictor, both: p < 0.001.*

The second strongest predictor was a high level of perceived stress in East Asia [β = 0.55, CI (0.22, 0.89), *p* < 0.001], North America [β = 0.44, CI (0.08, 0.79), *p* < 0.001], Southern Europe [β = 0.35, CI (0.21, 0.50), *p* < 0.001], Western Europe [β = 0.32, CI (0.15, 0.49), *p* < 0.001], Latin America [β = 0.24, CI (−0.09, 0.56), *p* < 0.001], and Eastern Europe [β = 0.23, CI (0.14, 0.43), *p* < 0.001]; and the third one was positive affect in Latin America [β = 0.29, CI (0.18, 0.40), *p* < 0.001], Southern Europe [β = 0.27, CI (0.23, 0.31), *p* < 0.001], North America [β = 0.24, CI (0.11, 0.38), *p* < 0.001], West Asia [β = 0.24, CI (0.16, 0.31), *p* < 0.001], East Asia [β = 0.24, CI (0.15, 0.33), *p* < 0.001], Eastern Europe [β = 0.23, CI (0.19, 0.28), *p* < 0.001] and Western Europe [β = 0.15, CI (0.10, 0.20), *p* < 0.001]. Lastly, psychological flexibility positively predicted prosocial behavior in East Asia [β = 0.18, CI (0.09, 0.27), *p* < 0.001], North America [β = 0.17, CI (0.04, 0.31), *p* < 0.001], Western Europe [β = 0.15, CI (0.12, 0.22), *p* < 0.001], Southern Europe [β = 0.11, CI (0.07, 0.16), *p* < 0.001], Latin America [β = 0.10, CI (0.00, 0.20), *p* < 0.001], and Eastern Europe [β = 0.07, CI (0.02, 0.12), *p* < 0.001] and Western Asia [β = 0.07, CI (0.00, 0.14), *p* < 0.001].

## Discussion

The COVID-19 pandemic fundamentally changed humans’ social lifes and day-to-day behaviors. The lockdown measures – imposed in many countries as a means to control the outbreak of the virus – was a concept-unheard of by most people at the time. Curfews, bans on gatherings, and the standstill of the public life dramatically impacted the frequency and quality of social interactions and resulted in the social isolation of many individuals. On the one hand, these restrictions conflict with the humans’ need to connect with others and to engage in positive social interactions, such as prosocial behavior. On the other hand, the severity of this crisis highlights the importance of prosocial behavior for a functioning society and the public mental health. This is why this study’s objective was to investigate the extent of prosocial behavior, its relation to well-being and factors predicting prosocial acts.

### Prosocial Behavior During the Pandemic

The present study revealed that, overall, prosocial behavior was reported to occur frequently during the first COVID-19 related lockdown in spring 2020. This finding largely supports the notion that in response to the social dilemma individuals do not shy away from supporting each other. Contrary to commonly held beliefs of panic and egoistic acts following disasters, humans tend to engage in various types of benevolent behaviors, as observed in diverse catastrophes (Zaki, 2020). Our results further indicated that helping behavior might occur universally given that the extent of reported prosocial behavior was comparable across eight regions covering 60 different countries around the world. Lower levels of prosocial behavior were observed in Eastern Europe and East Asia, which is likely explained by a combination of cultural, historic and political factors as well as country-specific regulations due to the pandemic. Indeed, when compared with Western families, traditional Asian families are more likely to emphasize family obligations and respect for hierarchical relations, implying that the prosocial tendencies are more likely to be displayed toward family members and peers (e.g., seniors), rather than other community members and strangers (Padilla-Walker et al., 2018). For Asian families, these motives may have been underrepresented in the Prosocialness Scale for Adults. On the other hand, lower levels of prosocial behavior in Eastern Europe compared to the overall sample might be explained by lower levels of social trust that has previously been observed in post-communist countries (e.g., Bjørnskov, 2007, 2021). If a society is characterized by doubts that most other people are behaving according to social norms, this might reduce the demonstration of prosocial behaviors. However, until replicated and specifically tested, these interpretations must remain speculative. Looking into specific types of prosocial behavior, our results show that spending time with friends who feel lonely and being available for volunteering were reported to occur the least frequently on average, a pattern observed across all regions. Given that state regulations involved restrictions drastically impacting social life, it is plausible that respondents refrained from spending time with friends. Research in a representative sample in the United Kingdom has shown that the number of daily contacts reduced substantially as a consequence of the physical distance measures in March 2020 (Jarvis et al., 2020). With regard to volunteering, it might be that this type of prosocial activity was less favorable due to a lack of volunteering options, or due to perceived danger in volunteering activities that involved social contact with others, as has been suggested in studies on informal and inexperienced volunteering (Whittaker et al., 2015). Another reason could be the lack of time and resources to commit to volunteering, particularly in working parents who were prone to experiencing high levels of distress due to the competing demands of childcare and employment as well as financial insecurity (Cheng et al., 2021).

### Relationship Between Prosocial Behavior and Well-Being

Importantly, we found that prosocial behavior was consistently associated with well-being across all regions, a finding consistent with a large body of evidence of a positive link between various types of prosocial behavior and well-being (Hui et al., 2020). One possible explanation of this result is that doing good to others feels good and that emotional rewards of helping are inherent to human nature. Previous studies established causal effects of prosocial acts on well-being (e.g., Martela and Ryan, 2016), with one study suggesting this phenomena to be a human universal, due to their finding that spending money on others leads to increases in well-being across cultures (Aknin et al., 2013). Our finding extends the current knowledge by indicating that the link between prosocial behavior and well-being is robust to the emotional and social intricacies of a global crisis. Due to the cross-sectional nature of this study, we cannot exclude the alternative explanation that individuals with high levels of well-being are more inclined to engage in prosocial behavior. However, there is preliminary causal evidence that generous actions during the pandemic result in positive affect, empathy, and social connectedness (Varma et al., 2020).

### Predictors of Prosocial Behavior

The present study examined sociodemographic and psychosocial predictors of prosocial behavior. Female gender, being retired, being unemployed, and living with parents were positively associated with prosocial behavior in the total sample. The role of retirement with regard to prosocial behavior has been studied extensively, as transitioning to retirement has been linked to increases in prosocial behavior (Fasbender et al., 2016). While previous research has primarily focused on volunteering in older age (Bjälkebring et al., 2021), our study suggests that retirement is positively related to prosocial behavior more broadly. Non-working populations might actively seek ways to be involved in social life, granting them opportunities to display prosocial behavior on the one hand, and satisfying psychological needs (i.e., meaning in life) on the other hand. With regard to unemployment, it is reasonable to assume that a shared understanding of being in need might facilitate prosocial behavior in this segment of the population. Previous research has produced cross-cultural evidence that individuals from lower socioeconomic backgrounds are more inclined to engage in prosocial behaviors compared to well-situated populations (Piff et al., 2010; Wang and Murnighan, 2014). Furthermore, in the context of the Spanish economic crisis, research showed that financial threat (i.e., due to unemployment) was related to increased helping behavior, suggesting that empathetic concern might give rise to prosocial actions (Alonso-Ferres et al., 2020). Similarly, those affected by unemployment during the COVID-19 pandemic might have been more prone to empathizing with and helping those in need. Interestingly, having an own family was negatively associated with engaging in prosocial acts. Given that a large proportion of our sample reported having children, this finding is likely explained by a lack of time parents are facing: The pandemic-induced governmental measures required working parents to navigate childcare (incl. home-schooling) and their work, oftentimes transferred to home-office, which put a tremendous burden on families and parents’ work-life-balance (Del Boca et al., 2020).

Prosocial behavior was most strongly associated with the perception of having social support in the overall sample, in particular high levels as compared to low levels of social support. Importantly, this finding was consistent across all regions, highlighting the central role of perceived social support with regard to benevolent actions independent of culture or society. Everyday helping behavior has been shown to occur more frequently with family and friends (Amato, 1990; Padilla-Walker and Carlo, 2014), with close others being the building blocks of a mutually supportive network. The availability of a caring and reliable network implicates positive interactions with strong social ties, which facilitates prosocial behavior (Barry and Wentzel, 2006). Perceived social stress, positive affect, and psychological flexibility were also connected to elevated prosocial behavior. The former might trigger prosocial behaviors due to a “tend-and-befriend”-response (Taylor et al., 2000), while a person who is more psychologically flexible may be able to temporarily disengage of his/her own emotions and focus on those in need of help (Chong et al., 2021).

With regard to regional variations, this study found similar patterns of meaningful predictors of prosocial behavior across the eight regions with one exception regarding psychosocial predictors worth mentioning: High levels of perceived stress were related to higher levels of reported prosocial behavior in all regions, except for Latin America, Western Asia, and Northern Europe. Based on the theoretical assumptions discussed in the introduction, one explanation could be that for individuals from these regions, prosocial actions are not employed as a way to regulate stress. Another explanation would be that engaging in prosocial behavior does not represent a source of stress in individuals from these regions, because prosocial acts might be culturally ingrained. It needs to be considered that these sub-samples are largely comprised of respondents from Colombia and Turkey, respectively, with societies that are characterized by collectivistic values and norms.

While psychosocial predictors showed comparable patterns across regions, sociodemographic variables gave a patchy picture when comparing regions. Some inconsistencies between regions merit further discussion: For example, unemployment was negatively related to prosocial behavior in Western Asia (consisting predominantly of Turkish respondents), whereas being unemployed predicted prosocial behavior in Northern Europe and Eastern Asia. While this finding might be explained by cultural differences, welfare-state measures existing in some countries might contribute to this finding. For example, the Nordic welfare states have a strong social support system and they have ensured easily accessible unemployment benefits as a response to the economic impact of the COVID-19 crisis (Greve et al., 2021). Experiencing financial security despite not having a job might put unemployed individuals emotionally, financially and time-wise in a situation that allows to start or continue an investment in prosocial behavior. Respondents from Western Asia, Eastern Europe and Northern America were also more inclined to prosocial behaviors when being affected by a worsening financial situation due to the pandemic (relative to facing a better financial situation), corresponding to the previous line or argumentation that economic downturn might give rise to prosocial responding in some regions. It should be noted that different sample sizes per region may also account for these differences in results.

The importance of these findings lay in the fact that prosocial behavior impacts individual well-being and society as a whole. Prosocial behaviors can drive meaningful change on a societal level by contributing to positive collective outcomes, such as resilience, solidarity and social connectedness in communities (Drury et al., 2009). Moreover, engaging in prosocial behavior might counteract adverse effects produced by the pandemic and related measures on the mental health and well-being of vulnerable groups. In the context of the COVID-19 pandemic, situations ranging from facing financial loss to being separated from loved ones pose an imminent threat to the mental health around the globe (Brooks et al., 2020; Luo et al., 2020). Importantly, social isolation and perceived loneliness were found to be strongly associated with depression, anxiety, self-harm and reduced well-being during the first lockdowns in spring 2020 (Gloster et al., 2020b; Luo et al., 2020), conditions that affected large parts of the population (Salari et al., 2020). Given the deleterious effects of the pandemic and associated measures on mental health, prosocial behavior has been suggested as a therapeutic target during the COVID-19 pandemic (Holmes et al., 2020), due to the positive effect it may exhibit on the mental health of both the providers and the receivers of support and help, with first intervention proposals addressing the impact of acts of kindness on mental health being on the way (Miles et al., 2021). Lastly, prosocial behavior has been discussed as a target for policy and intervention with regard to disease containment. Preliminary evidence demonstrates that prosocial emotions can be used as one path to instigate behavior change with studies showing that prosocial behavior is positively related to adhering to policy-relevant health behaviors including physical distancing, staying at home when sick, and adhering to hygiene recommendations (Pfattheicher et al., 2020; Campos-Mercade et al., 2021). Consistent with the idea of collective cooperation, a study on preventive actions demonstrates that people exhibit greater intend to engage in preventive efforts, such as distancing, when public health messages are framed as a way to help or protect others rather than appeals focused on the individual benefit of such behavior (Jordan et al., 2020).

Several limitations need to be acknowledged when interpreting the results of the current study: First, the cross-sectional study design did not allow for causal inferences nor for any accounts on the fluctuation of prosocial behavior across time. Second, we used a self-report measure of prosocial behavior, which might be prone to socially desirable reporting. While this is a common challenge in behavior research, anonymous survey administration could reduce the tendency of responding in a way that was viewed favorable by most societies. Additionally, using self-report measures was the only feasible way of assessing prosocial behavior in a large-scale, online survey. Third, despite the generally large sample size afforded by the wide reach of countries around the globe, in-country sample sizes varied substantially and were very small in some countries. While this problem was circumvented with the clustering of different countries into larger, geographically and culturally comparable regions, it needs to be considered that neither the regions were representative of included countries, nor the samples of each country.

## Conclusion

The pandemic-induced lockdowns served the goal of reducing the transmission of the COVID-19 virus. It simultaneously created social isolation, with undesirable impact on the public mental health globally. There has been a pressing need for social cohesion and helping behavior(s), likely influencing individual well-being. The findings of the present study are reassuring that even when experiencing complications of a global crisis, prosocial behavior consistently occurred across the world. Such behavior was associated with better well-being across all regions. Future policy efforts should create ways of incorporating the social network of a person and address malleable psychological competencies, in order to facilitate prosocial behavior in the process of fighting the spread of the virus.

## Data Availability Statement

The raw data supporting the conclusions of this article will be made available by the authors, without undue reservation.

## Ethics Statement

The studies involving human participants were reviewed and approved by Cyprus National Bioethics Committee. The patients/participants provided their written informed consent to participate in this study.

## Author Contributions

EH has conducted analysis. All authors contributed to the article and approved the submitted version.

## Conflict of Interest

The authors declare that the research was conducted in the absence of any commercial or financial relationships that could be construed as a potential conflict of interest.

## Publisher’s Note

All claims expressed in this article are solely those of the authors and do not necessarily represent those of their affiliated organizations, or those of the publisher, the editors and the reviewers. Any product that may be evaluated in this article, or claim that may be made by its manufacturer, is not guaranteed or endorsed by the publisher.
